# Assessing the impact of phosphodiesterase 5 inhibitors on survival in men after treatment for rectal cancer

**DOI:** 10.3389/fonc.2026.1940968

**Published:** 2026-09-22

**Authors:** Myroslav Lutsyk, Jonathan Green, Igor Albitskiy, Gil Bar-Sela

**Affiliations:** 1 Emek Medical Center, Oncology Institute, Radiation Therapy Department, Afula, Israel; 2 Bruce Rappaport Faculty of Medicine, Technion-Israel Institute of Technology, Haifa, Israel; 3 Statistics Department of Tel Aviv University, Tel-Aviv, Israel

**Keywords:** mortality risk, phosphodiesterase type 5 inhibitors, propensity score matching, rectal cancer, survival analysis

## Abstract

**Background/objectives:**

Phosphodiesterase type 5 inhibitors (PDE5Is) facilitate the attainment and maintenance of erections during sexual stimulation. Because erectile dysfunction is common after rectal cancer treatment, PDE5Is are frequently used in this population. We assessed whether PDE5I use was associated with overall survival after rectal cancer treatment. Methods: Utilizing data from Clalit Health Services in Israel, the analysis included 1,552 patients, with 256 users of PDE5Is. Survival differences were evaluated using categorical and non-parametric tests, Cox proportional hazards models, and propensity score matching to control for confounding variables.

**Results:**

PDE5I use was found to be significantly associated with improved survival, with a hazard ratio of 0.455 (p<0.001). The survival benefit persisted after adjusting for covariates and implementing matching procedures.

**Conclusions:**

These findings suggest a potential association consistent with a protective effect of PDE5I use as well as duration of use on survival in rectal cancer patients, warranting further investigation into underlying mechanisms and clinical implications.

## 1 Introduction

According to the World Health Organization’s International Agency for Research on Cancer, rectal cancer is the tenth most common cause of cancer-related death and ranks eighth in terms of cancer incidence worldwide; in Europe and the USA, it ranks respectively third and fourth (1). The survival rate for rectal cancer patients has significantly improved in recent years (2). In Israel, the survival rate for male rectal cancer patients is approximately 70% (3). Although the guidelines for rectal cancer treatment have changed over the years, the morbidity caused by surgery and chemoradiation remains a significant issue (4, 5). In addition to experiencing low anterior resection syndrome (fecal incontinence, frequency, urgency, or feelings of incomplete emptying), male patients also suffer from erectile dysfunction (ED) (6–8).

Over the past few decades, phosphodiesterase 5 (PDE5) inhibitors have revolutionized the treatment of erectile dysfunction, which is observed in about 52% of males between the ages of 40 and 70 (9). PDE5 is present in various tissues and is responsible for hydrolysis of cyclic adenosine monophosphate (cAMP) and cyclic guanosine monophosphate (cGMP) (10, 11). PDE5 is the primary enzyme responsible for breaking down cGMP in the corpus cavernosum (erectile tissue of the penis). PDE5 inhibitors (PDE5Is) block the degradative action of PDE5, which prevents the breakdown of cGMP, prolonging the effect of cGMP, enhancing and sustaining erection in response to sexual stimulation (12). PDE5 plays a crucial role in maintaining intracellular homeostasis and the PDE5 enzyme family is involved in inflammatory processes, immune responses, and tumor cell biology (13–16).

While PDE5Is were approved by the FDA decades ago for the treatment of erectile dysfunction, there is accumulating evidence of their anti-cancer/anti-tumoral activity. Colorectal tumorigenesis starts with the development of benign polyps. *In vivo* studies have shown that PDE5Is can reduce the rate of polyp formation by 50% (REF). PDE5Is have been shown to induce cell cycle arrest in colorectal cancer cell lines *in vitro*, ultimately leading to apoptosis (REF). Moreover, high expression of PDE5 itself in tumors often correlates with worse prognosis (17). In addition, PDE5Is increase the concentration of chemotherapeutic agents in tumor tissues (18).

As mentioned above, PDE5Is are widely prescribed to rectal cancer patients to manage treatment-related ED. This unique clinical context not only makes rectal cancer patients substantial real-world users of PDE5Is, but also presents an important opportunity to investigate whether these drugs confer additional survival benefits beyond symptomatic relief. Furthermore, since emerging preclinical and epidemiological evidence suggests that PDE5Is may have direct anti-tumor effects specific to colorectal neoplasms, including modulation of the tumor microenvironment and enhancement of immune responses, survival outcomes are particularly relevant and clinically meaningful endpoints to explore in rectal cancer patients using PDE5Is.

Clalit Health Services (CHS) provides health care to over 52% of Israel’s 9.67 million citizens (19). CHS data, managed via MDClone, integrates clinical information from community clinics, hospitals, and emergency departments, providing a comprehensive national dataset (20). Access to this data enabled us to conduct a retrospective study to evaluate the potential correlations between the use of PDE5Is and survival improvements after completion of rectal cancer treatment.

The aim of the study was to assess, in adults with rectal cancer treated within CHS, whether use of PDE5Is is associated with an overall survival advantage over patients who did not use PDE5Is during long-term follow-up, using multivariable and propensity-score–based analyses. We focused on overall survival among men with rectal cancer treated in a real-world setting, rather than broader colorectal cancer populations or mixed indications for PDE5 inhibitor use. By leveraging a large, population-based dataset from Clalit Health Services, our analysis provides one of the first detailed evaluations of the association between PDE5I use and survival specifically in rectal cancer survivors, thereby helping to bridge the gap between mechanistic/preclinical data and clinically observed outcomes in this distinct patient group.

## 2 Materials and methods

### 2.1 Study sample

In Israel, CHS is the largest of four primary healthcare providers. CHS data, managed via MDClone, integrates clinical information from community clinics, hospitals, and emergency departments, providing a comprehensive national dataset (20). According the National Insurance Agency, in 2024 CHS provided health care to over 52% of Israel’s 9.67 million citizens (19). The CHS offers researchers access to a scientific platform called the “Research Room,” which serves as a data source for the current study. According to current regulations, all medical data have to be anonymized. The data had been pre-filtered according to predefined inclusion and exclusion criteria. The final sample was comprised of 1,552 patients, of whom 256 had purchased PDE5Is as sexual performance enhancement drugs at some point. The patients’ ages at diagnosis had mean 65.12 ± 12.80 (range, 26–95 years).

### 2.2 Data collection and processing

The anonymizing level chosen for the current study required the removal of detailed descriptions, including the type and exact date of surgery, the place and date of radiotherapy, and the precise chemotherapy protocol, as well as the stage of disease at the time of diagnosis, from the database. Variables with no variation or many missing values were also removed.

### 2.3 Patient selection and inclusion criteria

In this retrospective study, we included individuals assigned male at birth who were diagnosed with non-metastatic rectal adenocarcinoma between January 2009 and October 2024. The treatment guidelines in Israel closely resemble those in the USA and European Community, with the recommendation of 5-FU-based chemotherapy and radiotherapy as neo-adjuvant and adjuvant therapy, according to clinical and/or pathological findings. Patients who were managed with a total neoadjuvant approach and did not undergo surgery were excluded due to the short follow-up time. The date of surgery was used as the initial time point for calculating PDE5I exposure time, as surgical resection is the primary curative treatment for rectal cancer.

Patients were distributed into two groups: users and non-users (defined by an active prescription for PDE5Is at the time of surgery or after). Patients who took the drug before surgery but did not continue after were combined with non-users, since we were assessing the effect of PDE5I on survival after surgery. Evaluating the impact of PDE5Is during pre-rectal cancer conditions is out of scope for the study. A study flow-diagram of cohort selection can be seen in **Figure 1**.

**Figure 1:**
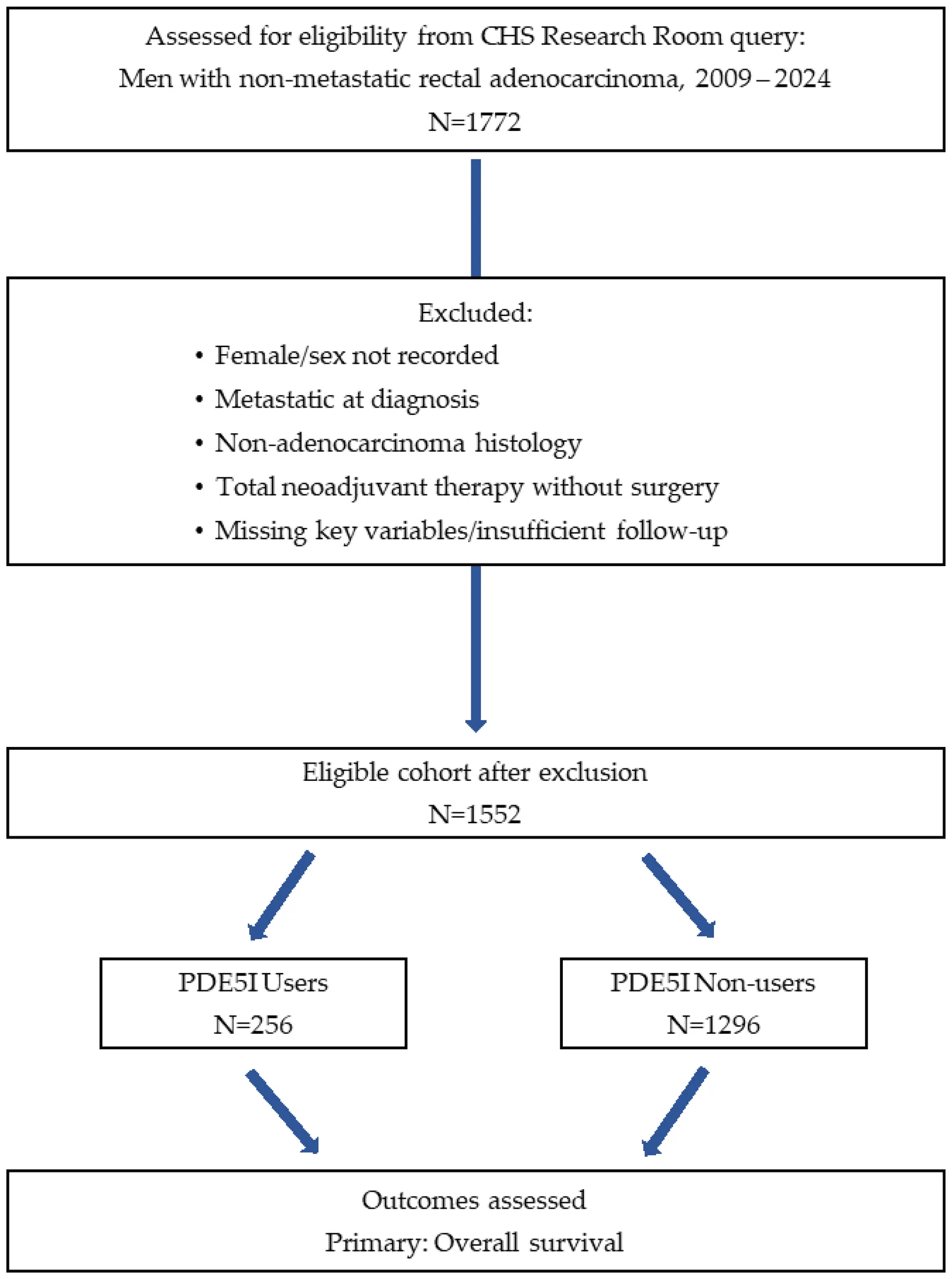
Study flow – cohort selection and exposure.

### 2.4 Ethnicity

In Israel, the population consists of Jews, Arabs, and other minority ethnic groups, each with distinctive cultural and religious backgrounds that may influence healthcare practices, including the use of PDE5Is.

### 2.5 Covariates

The independent variables used in the current study included both demographic and clinical features, which were mostly categorical, with a few numeric exceptions. Demographic variables include socio-economic status and ethnicity. Socioeconomic status was included because it may be associated with healthcare utilization, treatment-seeking behavior, and long-term outcomes; notably, it was significantly associated with survival in the multivariable Cox PH model. Clinical covariates include diagnosis age, surgery year, PDE5I use (modeled as a fixed variable), age of PDE5I use onset (set to 0 for non-users), Charlson Comorbidity Index (CCI) score, and individual (CCI) conditions. The AIDS indicator, which had no variance, was removed. An indicator for connective-tissue disease was also removed due to missing data in over 50% of patients. No additional variables had missing values. The use of PDE5Is was based on data on purchases and dosages obtained from the CHS database. Since various drugs with similar bioeffects are available on the Israeli market, the minimal effective dose of sildenafil (50 mg) was used as the “active unit” for comparing the consumption rates of similar medications in this class. Exposure time was not included in the dataset as temporal analyses are outside the scope of the current study. The final dataset consisted of a total of 26 covariates (independent variables).

### 2.6 Time-to-event variables

The time-to-event variables (which serve as the dependent variables in survival analyses) consisted of an observed survival variable (measured in years from time of surgery) and an event indicator (death = 1, censored = 0).

### 2.7 Statistical methods

#### 2.7.1 Categorical and non-parametric analyses

Differences between users and non-users were examined with respect to demographic and clinical attributes. These were performed using Chi-squared tests for categorical variables and Wilcoxon rank-sum tests for numeric variables for which normality could not be assumed. Normality of numeric variables within PDE5I use levels was assessed via Shapiro-Wilks normality tests.

#### 2.7.2 Initial survival analysis

We began our analysis with a log-rank test comparing survival distributions between PDE5I users and non-users. Next, we fit a Cox proportional hazards (PH) model including all available covariates to assess the variables that significantly influence survival, as well whether the effect of PDE5I use remains significant after adjusting for other covariates.

#### 2.7.3 Addressing confounding with propensity score matching

To reduce the impact of potential confounding, we employed Propensity Score Matching (PSM). The covariates to be balanced were selected by extracting the most prognostic variables in terms of survival outcome, using two variable selection methods:

1. A standardized Cox PH model (with all numerical covariates set to have 0 mean and unit variance).
2. A Random Survival Forest (RSF), using 10,000 trees, log-rank splitting criteria, and a minimum terminal node size of 10. Bootstrap sampling was used for observations, and model performance was assessed using out-of-bag (OOB) data at each RSF iteration. Variables were subsampled at each split by the default method (number of covariates divided by three). The RSF achieved a mean OOB C-index of 0.754 with SD = 0.0015.

Variable importance was extracted from the RSF by comparing the model’s splits with random splits. For the Cox PH model, all numerical covariates were standardized to allow comparison of effect sizes. Variables with the largest standardized coefficients (in absolute value) were interpreted as those most strongly associated with survival.

We selected the top six variables from both models (resulting in a total of seven variables due to overlap), based on their consistency across both variable selection methods as well as their clinical interpretation. The ordered normalized importance of these variables can be found in the **Supplementary Material** (**Supplementary Table 1**). We then applied PSM, treating PDE5I use as the treatment indicator. The covariates used for matching were: ethnicity, age at diagnosis, recurrence (inferred from medical records), CCI score, comorbidity count, and socioeconomic status (added since it exhibited significance in the Cox PH model), as well as the presence of “liver disease” and “malignancy” in the CCI.

We evaluated several matching ratios and caliper combinations. The optimal balance was achieved with a 1:3 user-to-non-user ratio, with caliper value 0.2. Balance was evaluated by comparing mean standardized differences or differences in proportions, for numeric and categorical variables, respectively. Matching was performed using the nearest neighbor approach without replacement.

The final matched dataset included 253 PDE5I users (3 unmatched) and 630 non-users. All eight covariates used for matching achieved mean standardized differences < 0.1. unadjusted and adjusted balance diagnostics are displayed in **Figure 2**.

**Figure 2:**
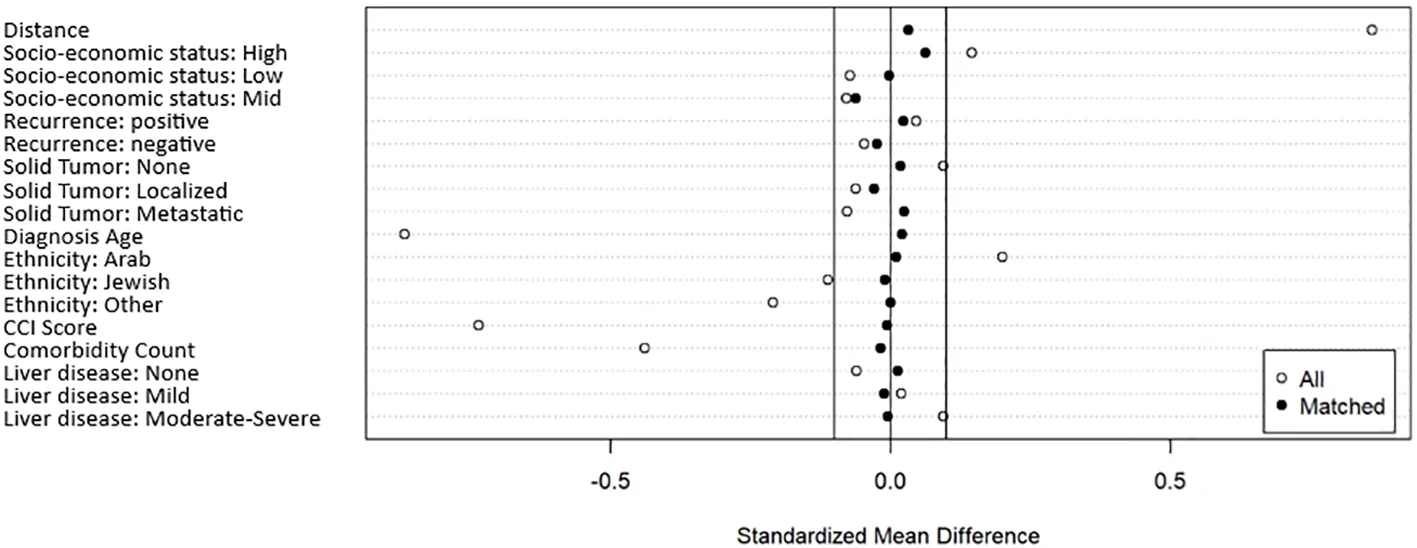
Standardized mean differences – unadjusted and adjusted using PSM with 1:3 User to Non-User Ratio and 0.2 Caliper. Standardized mean differences pre- and post-PSM, using our final PSM algorithm. The solid lines represent the 0.1 threshold. All matched standardized mean differences are below the 0.1 threshold (in absolute value).

To assess whether the effect of PDE5I use on survival persisted after covariate balancing, we re-ran the log-rank test on the matched dataset. In addition, we reran the Cox-PH model to explore other effects, such as duration of exposure among users.

#### 2.7.4 Additional analyses

In order to assess whether **t**he survival differences are consistent within subgroups of categorical variables and to examine potential interactions, we conducted stratified log-rank tests by dividing the full dataset into binary levels of ethnicity (Arab and Jewish) and disease recurrence (recurrence and no recurrence).

P-values are reported for all statistical tests. Key findings are presented graphically. All analyses were conducted in R (version 4.2.3).

## 3 Results

### 3.1 Descriptive statistics of patient data

The CHS database contained information on 1,552 patients diagnosed with non-metastatic rectal cancer at the time of their diagnosis. The patient characteristics are presented in **Table 1**. The drug exposure mean time was 4.71years (95% CI 4.14–5.29).

**Table 1:** Summary of analysis of users versus non-users demographic and clinical attributes.

Demographic and clinical attributes		Non-users	Users	Total	χ² test, (df)	p-value
Ethnicity	Arabs	159 (12.3%)	52 (20.3%)	211	19.77, (2 df)	p<0.001
	Jews	1091 (84.2%)	204 (79.7%)	1295		
	Other	46 (3.5%)	0	46		
Socioeconomic Status	High	400 (30.8%)	97 (37.9%)	497	4.849 (2 df)	0.088
	Low	433 (35.8%)	77 (30.1%)	510		
	Mid	463 (33.4%)	82 (32.0%)	545		
Age at Diagnosis Mean ± SD		66.6± 12.6	57.4 ± 10.6	65.1 ± 12.8	239625	p<0.001
Recurrence	Yes	324 (25.0%)	59 (23.0%)	383	0.34, (1 df)	0.560
	No	972 (75.0%)	197 (77.0%)	1169		
Radiotherapy	Yes	592 (45.7%)	146 (57.0%)	738	10.6, (1 df)	p<0.01
	No	704 (54.3%)	110 (43.0%)	814		
Tumor location	Rectum	1041 (80.3%)	213 (83.2%)	1254	0.96, (1 df)	0.326
	Rectosigmoid	255 (19.7%)	43 (16.8%)	298		
CCI Score Mean ± SD		5.11 ± 2.9	3.3 ± 2.4	4.81 ± 2.9	228724	p<0.001
Surgery Year Mean ± SD		2014.97 ± 3.82	2014.55 ± 3.85	2014.90 ± 3.82	176337	0.109

Our categorical and non-parametric analyses revealed significant associations between PDE5I use and ethnicity (χ² = 19.778, 2 df, p < 0.001) and age at diagnosis (Wilcoxon rank-sum test: W = 239625, p < 0.001, effect size = 0.286). Users and non-users did not significantly differ in terms of socioeconomic status. Among the PDE5I users (n=256), 204 (79.7%) were Jewish, while 54 (20.3%) were Arabs. This compared to 1091 Jews (84.2%), 159 Arabs (12.3%), and 46 (3.5%) belonging to some other ethnic minority, among non-PDE5I users. Users and non-users also differed significantly in age at diagnosis, with users (mean age = 57.4 ± 10.6 years) tending to be younger than non-users (mean = 66.6± 12.6 years).

Regarding clinical attributes, we observed a significant effect for radiotherapy (RT) (χ² = 10.6, p < 0.01), whereby 592 non-users (45.7%) received RT, compared to 146 (57%) of users. Users and non-users also differed significantly in terms of CCI scores. Non-users had a mean score of 5.11 ± 2.97, as opposed to 3.32 ± 2.42 among users (Wilcoxon rank-sum test: W = 228724, p < 0.001, effect size = 0.245). Users and non-users did not significantly differ in terms of recurrence rate or tumor location. Full statistical summaries of the comparison of patients’ demographic and clinical attributes, as well as Shapiro-Wilks normality assessments of numeric attributes, are provided in **Tables 1** and **2**. Distributions of patients by age at diagnosis can be found in the **Supplementary Material** (**Supplementary Figure 1**).

**Table 2:** Normality assessments of users’ and non-users’ numeric attributes by Shapiro-Wilk test.

Group/comparison	Statistic	p-value	Interpretation
Age – users	0.992	0.176	Normality not rejected
Age – non-users	0.981	p<0.001	Normality rejected
CCI score – users	0.902	p<0.001	Normality rejected
CCI score – non-users	0.959	p<0.001	Normality rejected
Surgery year – users	0.947	p<0.001	Normality rejected
Surgery year – non-users	0.949	p<0.001	Normality rejected

### 3.2 Initial survival analysis

The initial log-rank test comparing survival between users and non-users revealed significantly better survival for users (22.66% deaths versus 53.94% among non-users). We obtained a Chi-squared test statistic value of 94.7, with p-value<0.001. The Observed-Expected table is presented below (**Table 3A**). A complete Cox PH model was fitted with all covariates. We obtained a Likelihood Ratio Test of 713.8 (32 df, n = 1552), with a p-value of <0.001. The total number of deaths was 757.

**Table 3:** Observed versus expected deaths among PDE5I users and non-users, whole population (A) and post-matching analysis (B).

PDE5I Use	N	Observed	Expected
Whole sample (A)			
Non-users	1296	699 (53.94%)	588.994
Users	256	58 (22.66%)	168.006
Post-matching analysis **(B)**			
Non-users	630	244 (38.7%)	198.478
Users	253	57 (22.5%)	102.522

Several covariates, including the use of PDE5Is, demonstrated statistically significant associations with survival. We report here the significant p-values and hazard ratios. Ethnicity was significantly associated with survival outcomes. Patients identified as non-Jewish/Arab exhibited a substantially higher hazard compared to the Arab reference group (HR = 23.738, p < 0.001). These patients may belong to peripheral or marginalized social groups and may face systemic healthcare boundaries. Disease recurrence was also a strong predictor of poorer survival outcomes (HR = 2.94, p < 0.001). Older age at diagnosis was associated with a modest but significant increase in hazard (HR = 1.044 per year, p < 0.001), which corresponds to a 4.4% risk increase per additional year. Lower socio-economic status was also linked to higher hazard (HR = 1.357, p < 0.01). Finally, PDE5I use was associated with a significantly reduced hazard compared to non-use (HR = 0.456, p < 0.001). This implies an approximate 54% reduction in instantaneous risk over time for PDE5I users compared to non-users. Full model estimates are provided in **Table 4**.

**Table 4:** Exponentiated results of full Cox PH model*.

Term	Estimate	S.E.	Statistic	p value	Significance
Ethnicity: Jewish	1.260	0.138	1.674	0.094	
Ethnicity: Other	23.738	0.229	13.856	p<0.001	***
Recurrence: positive	2.940	0.080	13.464	p<0.001	***
Diagnosis Age	1.044	0.011	3.769	p<0.001	***
Drug use onset age	1.000	0.002	0.143	0.886	
Drug use: positive	0.456	0.167	-4.712	p<0.001	***
Radio-therapy: positive	0.944	0.079	-0.734	0.463	
Years of Drug Use	0.973	0.020	-1.413	0.158	
Surgery Year	1.014	0.012	1.203	0.229	
Socio-economic status: Low	1.357	0.099	3.095	p<0.01	**
Socio-economic status: Mid	1.185	0.094	1.810	0.070	
Tumor Location: Rectum	0.852	0.095	-1.679	0.0931	
CCI Score	0.915	0.116	-0.763	0.445	
Comorbidities Count	1.291	0.263	0.970	0.332	
Cerebral-vascular disease: positive	0.962	0.264	-0.148	0.883	
Chronic-pulmonary disease: positive	1.058	0.264	0.213	0.831	
Congestive Heart Failure: positive	1.132	0.269	0.462	0.644	
Dementia: positive	1.153	0.289	0.493	0.622	
Diabetes Mellites: Uncomplicated	0.819	0.252	-0.792	0.428	
Diabetes Mellites: End-organ Damage	1.347	0.300	0.992	0.321	
Hemiplegia: positive	1.471	0.356	1.082	0.279	
Leukemia: positive	0.730	0.511	-0.615	0.538	
Liver disease: Mild	0.753	0.287	-0.989	0.322	
Liver disease: Moderate-Severe	1.982	0.513	1.334	0.182	
Lymphoma: positive	1.380	0.391	0.822	0.411	
Solid Tumor: Localized	0.941	0.276	-0.222	0.824	
Solid Tumor: Metastatic	2.985	0.643	1.700	0.089	
Myocardial Infraction: positive	0.775	0.265	-0.963	0.335	
Peptic-ulcer disease: positive	0.944	0.278	-0.206	0.837	
Peripheral Vascular disease: positive	1.050	0.264	0.183	0.855	
Chronic Kidney disease: positive	1.466	0.296	1.291	0.197	

*The table displays the full Cox PH model. For each variable, the exponentiated coefficient, standard error, statistic and p-value are reported. The last column denotes levels of significance: *<0.05, **<0.01 and ***<0.001. Variables with significant effects include: ethnicity, recurrence, PDE5I use, age at diagnosis and socioeconomic status.

### 3.3 Post matching analysis

To assess whether the effect of PDE5I use on patient survival remained significant after balancing the data concerning the most informative variables, we refit the log-rank test to the matched data. Indeed, this confirmed that PDE5I users continued to show significantly prolonged survival: χ² = 31.08973, p-value < 0.001. The Observed-Expected Table is presented below (**Table 3B**). The Hazard Ratio was extracted from a Cox PH model, this time fitted post-PSM (Likelihood Ratio Test of 305.5 on 29 df, n = 883, p-value <0.001). We obtained HR = 0.361, p<0.001, which corresponds to an instantaneous survival risk reduction of about 64% among PDE5I users compared to non-users. For Years of PDE5I use we obtained a HR = 0.914, p<0.01. This corresponds to an 8.6% survival risk reduction per each year of PDE5I use. The Kaplan-Meier survival curves for pre- and post-PSM cohorts are shown in **Figures 3** and **4**. The median survival time for non-users, using the original data, was 7.3 years, whereas after balancing the data, it increased to 13.5 years. For users, the median survival time was not reached in either analysis.

**Figure 3:**
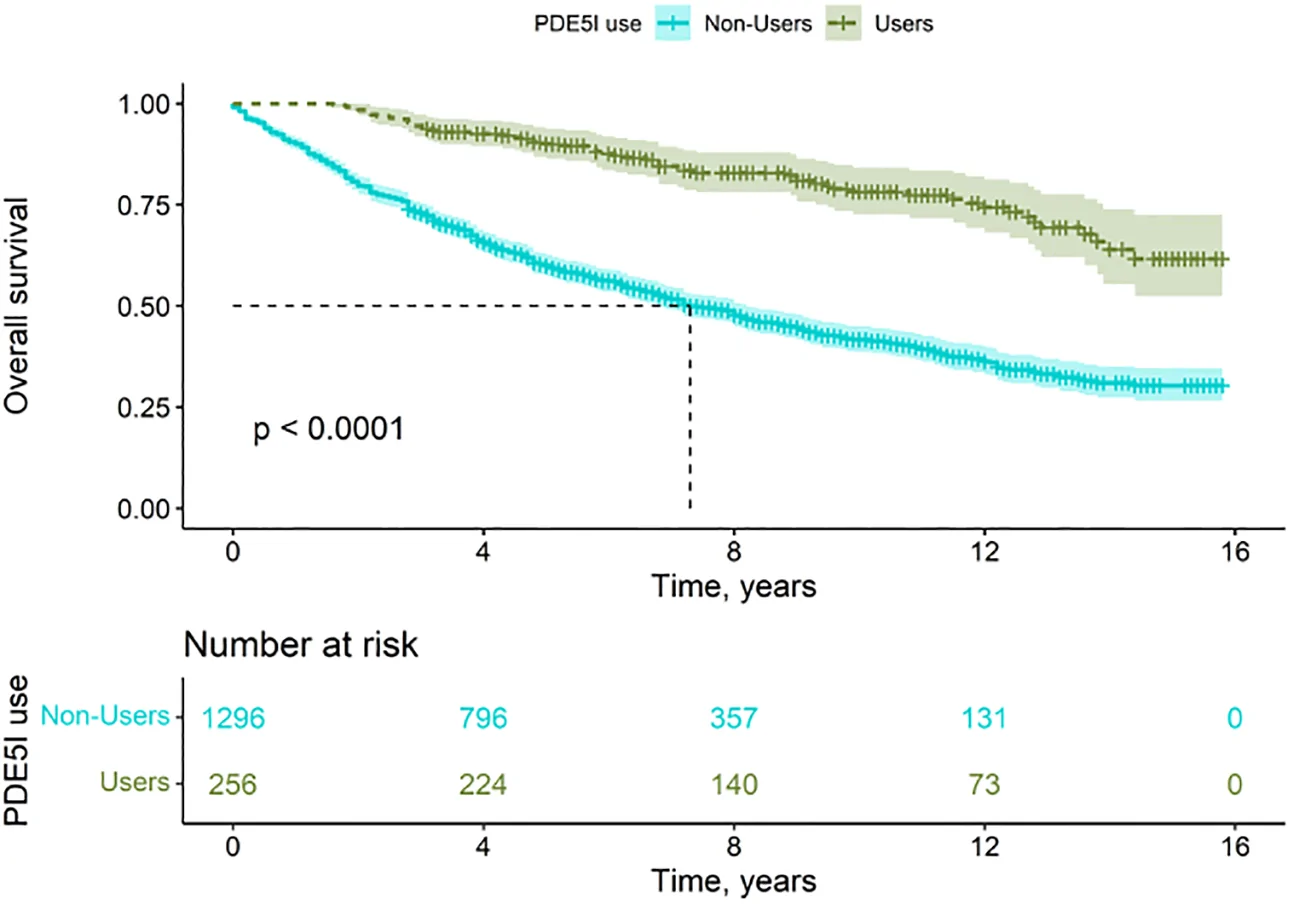
KM survival estimates for PDE5I users versus non-users (unadjusted). Green represents users, while blue represents non-users. The plot also displays the log-rank test p-value, which is significant given any reasonable significance level. The vertical and horizontal dashed lines in the graph represent the median survival for non-users. Median survival was not reached for PDE5I users.

**Figure 4:**
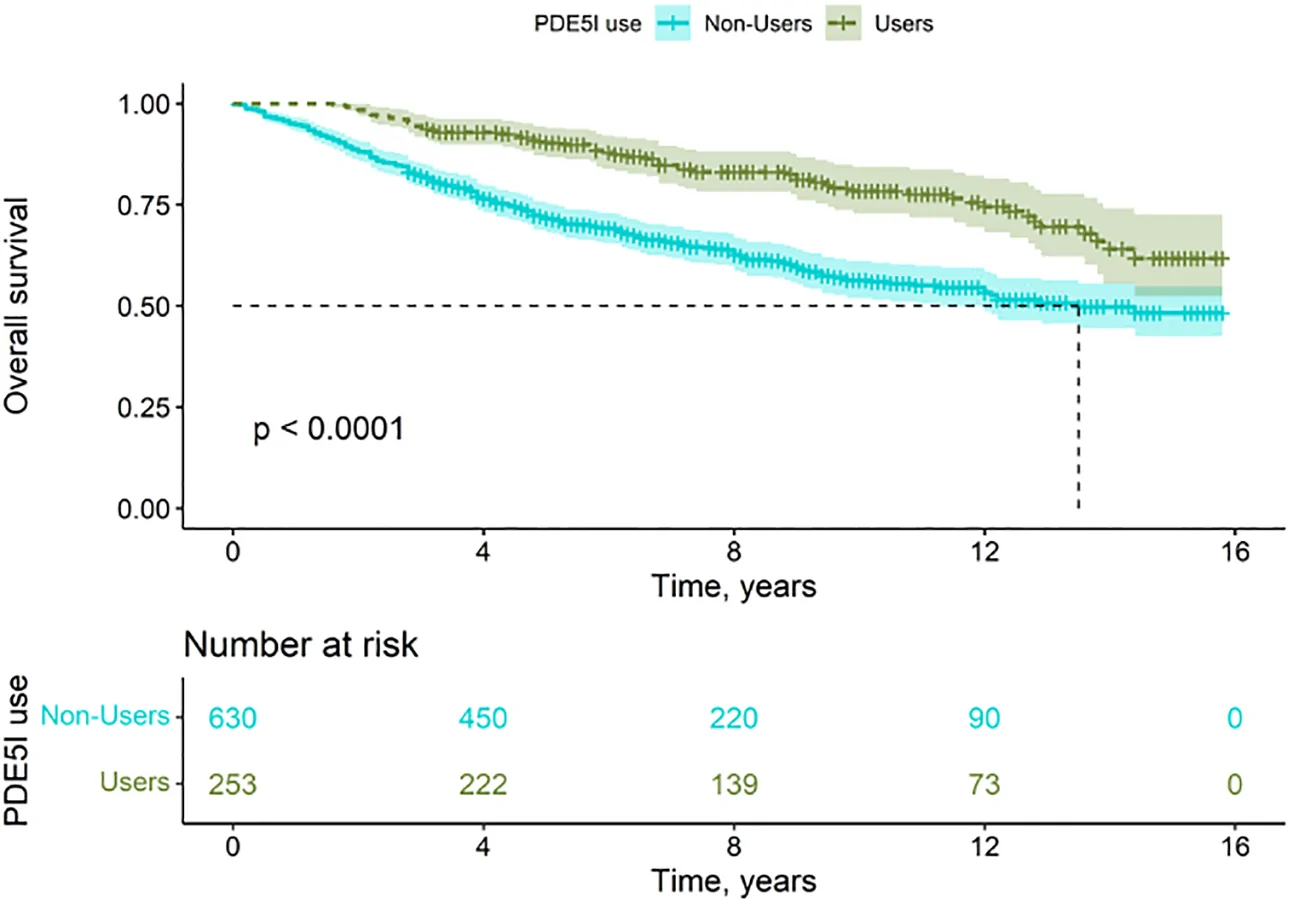
KM survival estimates for PDE5I users versus non-users (adjusted with PSM). Green represents users, while blue represents non-users. The plot also displays the log-rank test p-value, which is significant given any reasonable significance level. The vertical and horizontal dashed lines in the graph represent the median survival for non-users. Median survival was not reached for PDE5I users. Although the effect was reduced after matching, a clear and significant difference remains.

### 3.4 Additional analyses

All stratified log-rank tests conducted on subgroups, defined by ethnicity and disease recurrence status, showed significant differences between users and non-users, with better survival rates for PDE5I users. The results are summarized in **Table 5**. Among patients with recurrence, we observed 259 events in non-users (79.9%) compared to 32 events in users (54.2%) (Chi-squared = 30.148, p < 0.001). Similarly, among patients without recurrence, the observed number of events was 440 (45.3%) for non-users and 26 (13.2%) among users (χ² = 73.576, p < 0.001). For Jews, we identified 1091 events among non-users (54.4%) and 47 events (23%) among users (Chi-squared = 80.756, p < 0.001). Among the examined subsamples, the least significant effect was observed among Arab patients, with 59 (37.1%) and 11 (21.1%) events for non-users and users, respectively (χ² = 4.869, p = 0.027).

**Table 5:** Summary of stratified log-rank tests performed for ethnicity and recurrence subgroups.

Subgroup	PDE5I Use	N	Observed	Expected	Chi-squared statistic	p-value
Recurrence	Non-users	324	259 (79.9%)	220.206	30.148	p<0.001
	Users	59	32 (54.2%)	70.794		
No recurrence	Non-users	972	440 (45.3%)	364.197	73.576	p<0.001
	Users	197	26 (13.2%)	101.802		
Arabs	Non-users	159	59 (37.1%)	50.787	4.869	0.027
	Users	52	11 (21.1%)	19.213		
Jews	Non-users	1091	594 (54.4%)	501.4	80.756	p<0.001
	Users	204	47 (23%)	139.6		

## 4 Discussion

Our retrospective study used the CHS database in Israel to examine the outcomes of 1,552 patients with rectal cancer, 256 of whom had purchased PDE5Is as sexual performance enhancement drugs post-surgery. Initial survival *analyse*s suggested that PDE5I use and duration of PDE5I use were associated with significantly better survival. This association persisted after adjusting for available potential confounders. Stratified log-rank tests on subgroups defined by ethnicity and recurrence status consistently showed better survival rates for users (p<0.001), except *among* Arab patients, where the association was less pronounced (p=0.027). This can likely be explained by the small sample size (only 14% of the patients were Arabs and of these only 20% were users), but it is also possible that differences in healthcare access, comorbidities, or cultural factors *play a part.*

Our findings support those of several other studies into the possible anticancer effects of PDE5Is. A large Swedish nationwide observational study revealed a significantly lower risk of colorectal cancer-specific mortality and reduced risk of distant metastases in PDE5I users versus non-users (21). The protective effect was notably stronger among patients who underwent open surgery, supporting a role for PDE5Is in counteracting surgery-induced immune suppression and improving oncologic outcomes. A retrospective, matched cohort study analyzing data from 5,545 prostate cancer patients who underwent prostatectomy found that PDE5I administration was associated with improved overall survival and reduced risk of death, suggesting routine use of PDE5I after prostatectomy may improve survival in prostate cancer patients (22). Another population-based cohort study from Sweden focusing on gastric cancer patients showed that PDE5I use was linked to lower cancer-specific mortality, particularly in early-stage cancers (23). While direct randomized clinical trials of PDE5Is in rectal cancer are still lacking, these population-based studies coupled with preclinical evidence provide convergent support for the potential adjuvant value of PDE5Is in colorectal malignancies, including rectal cancer.

Our observed association was larger than that reported in previous colorectal cancer studies. Huang et al. (2020) reported a 18% reduction in colorectal cancer-specific mortality (HR 0.82) among PDE5I users. The larger effect size in our study (HR 0.455; median survival difference of ~6 years) may reflect differences in population (rectal cancer survivors vs. broader CRC cohorts), exposure timing (post-treatment vs. any post-diagnosis), outcome definition (overall vs. cancer-specific survival), and adjustment strategies. This magnitude also raises the possibility that residual confounding or healthy-user bias may inflate the observed association beyond a true drug effect. Some likely potential confounders which were not taken into account in the current study are cancer stage, BMI and smoking status. In addition, modelling PDE5I using as a time fixed variable could have inflated the observed effect due to the immortal-time bias. The post-matching analysis revealed a more conservative HR of 0.914 for the duration PDE5I use (in years, post-surgery), which corresponds to a statistically significant 8.6% risk reduction for each additional year of PDE5I use. This finding may be more interpretable as it is not affected by the immortal-time bias.

Several potential mechanisms have been proposed to explain the observed association between PDE5I use and survival. Emerging evidence indicates a duality in PDE5Is’ effects on tumorigenesis and oncoprotection in different sites and organs, as presented in a recent review (14).

Overall, the hypotheses regarding the contribution PDE5I to the survival of cancer patients can be divided into two approaches. The first is that the same mechanism that leads to improved erectile function could also contribute to improved survival; increasing blood flow to the pelvis improves tissue oxygenation, reduces inflammation and improves healing after oncological surgeries and radiotherapy. This has been suggested as the cause of the anti-inflammatory effects observed in prostate tissue (15). Increased blood flow to the tumor area also improves accessibility of anti-tumor drugs to their target, so improving the survival of those patients receiving chemotherapy (24). Adequate oxygenation in the tumor microenvironment is a well-established factor in enhancing the cytotoxic efficacy of radiation as well as the response to cytotoxic drugs (25). Other effects include inhibition of efflux pumps within tumor vasculature. PDE5 inhibitors decrease efflux pump activity, enhancing the EPR effect, thereby increasing the concentration of the anticancer drug in tumor tissues (26). The second approach explains the clinical findings through more direct anti-cancer effects via molecular pathways involving cGMP or other molecular mechanisms – such as PDL1 upregulation, cell cycle arrest, and induction of apoptosis (27, 28). One major mechanistic pathway involves immunomodulation after oncologic surgery. Preclinical and clinical data reveal that cancer surgery induces a state of immunosuppression, with a prominent expansion of myeloid-derived suppressor cells (MDSCs). These MDSCs inhibit cytotoxic T lymphocyte (CTL) and natural killer (NK) cell functions, impairing tumor clearance and promoting distant metastasis. PDE5Is such as sildenafil have been shown in preclinical and early clinical studies to block MDSC recruitment and restore CTL and NK cell activity, reducing surgery-induced immune paralysis and lowering metastasis rates. This immunomodulatory effect is supported by observations that PDE5I users experience significantly lower risks of post-operative metastasis and cancer-specific mortality (17).

PDE5Is also impact tumor cell biology at the molecular level. Zhang’s study mentioned above combined their population-based evidence with *in vivo* and *in vitro* experiments to investigate the mechanisms by which the PDE5I sildenafil suppresses gastric cancer growth (23). They found that by inhibiting the hydrolytic activity of PDE5, sildenafil increases intracellular cGMP, which activates protein kinase G. This leads to cell cycle arrest, increased apoptosis, and degradation of oncogenic factors such as c-MYC via downstream suppression of IL-6/JAK/STAT3 signaling. Another possible mechanism for the effect on cell cycle is an increase in levels of reactive oxidative species, which in turn impairs the function of CDK and PARP proteins (29). It is worth noting that some evidence suggests patients taking PDE5Is may have a higher risk of developing melanoma, which is linked to the activation of the RAS/RAF/ERK signaling pathway in melanoma cells (30). However, a recent Mendelian randomization analysis found little evidence to support associations between genetically proxied PDE5 inhibition and 20 cancers. They did identify a protective effect of genetically proxied PDE5 inhibition on colorectal and gastric cancer risk (31).

Preclinical studies corroborate these mechanisms in colorectal cancer models. *In vitro*, PDE5Is inhibit proliferation and trigger apoptosis in colorectal cancer cell lines with IC50 values within achievable pharmacologic ranges (29). *In vivo*, oral PDE5Is reduce polyp formation and tumor growth by 50-60% in chemically-induced murine models through reduction of inflammation and MDSC infiltration (32). Genetic data from The Cancer Genome Atlas (TCGA) indicates that high PDE5 expression in colorectal tumors correlates with poorer survival, underscoring PDE5 as a viable therapeutic target (17). Together, these preclinical and translational data underscore the clinical relevance of PDE5Is’ direct molecular and immunomodulatory actions in colorectal and consequently rectal tumors.

Overall, the findings of our study are consistent with a potential role of PDE5 inhibitors as adjunctive agents in the management of rectal cancer, but they should be interpreted as hypothesis-generating rather than definitive evidence of benefit. This option has been explored *in silico* with a novel, more potent PDE5 inhibitor, which had greater apoptotic effects and higher efficacy as an anti-tumor agent (33). More recently, Ariav et al. identified an additional non-canonical mechanism by which PDE5A inhibition may restrict cancer progression (34). Sildenafil-induced elevation of intracellular cGMP was shown to impair NPC1-mediated lysosomal cholesterol export, resulting in reduced cellular cholesterol availability, disruption of membrane lipid rafts and mitochondrial bioenergetics, and suppression of cancer-cell migration and metastatic capacity. Importantly, these effects were demonstrated across several experimental models, including human colon cancer cells and murine colon cancer models. The same study also reported improved survival associated with sildenafil exposure in analyses of electronic health records. These findings provide a biologically plausible mechanistic framework for the association observed in our cohort, although our retrospective clinical data cannot establish that this pathway accounts for the survival difference.

Nevertheless, these mechanistic observations do not resolve the important limitations inherent to observational clinical data. This study also has several important strengths that support the validity and clinical relevance of the findings. It utilized a large, nationally representative dataset from CHS, Israel’s largest healthcare provider, enabling robust statistical power and generalizability to diverse patient populations. The comprehensive linked data allowed detailed longitudinal follow-up and adjustment for numerous demographic, clinical, and treatment covariates, reducing confounding bias.

The study focused on rectal cancer patients, a population with high morbidity and mortality in which PDE5I use for postoperative erectile dysfunction is common. This real-world setting supports the clinical applicability of the findings. The broadly consistent survival benefit seen across subgroups defined by ethnicity and disease recurrence status suggests a robust effect of PDE5Is in improving rectal cancer prognosis.

This study has several limitations, including potential residual confounding despite the use of propensity score matching, which may not fully account for unmeasured variables influencing survival outcomes, specifically cancer stage. In particular, PDE5I users more frequently received radiotherapy - itself an established risk factor for erectile dysfunction - so differential radiotherapy exposure may increase the likelihood of subsequent PDE5I use and represents a relevant potential confounder, which we addressed by including radiotherapy as a covariate in the multivariable model. Furthermore, the immortal-time bias could partially explain the extremity of the HR we obtained for PDE5I use, as it was modeled as a time-fixed variable. The observational design precludes establishing causality between PDE5I use and improved survival, and there may be selection bias related to patients who purchase these medications, such as differences in health behavior or access to healthcare. The anonymized Research Room dataset did not reliably identify the prescribing physician’s specialty or whether the patient received specific counseling from a urologist or another healthcare professional about PDE5I administration. Pharmacy records document dispensing rather than ingestion and do not provide reliable information on treatment frequency, on-demand versus scheduled use, missed doses, treatment discontinuation, adherence, or adverse effects; these factors add another limitation. One important concern is healthy user bias: patients prescribed PDE5Is may represent a subgroup of healthier, more health-conscious men with better baseline health status and healthcare engagement. This could partially explain the survival differences observed, independent of any direct biological effect of the drug. Patients actively seeking treatment for erectile dysfunction might also have better overall postoperative recovery or adherence to oncologic follow-up and adjuvant therapies. Though propensity score matching and multivariable adjustments were used to reduce confounding, unmeasured factors such as lifestyle, frailty, and socioeconomic status may still influence the association. In addition, data on smoking status, obesity or body-mass index, and baseline erectile function were not reliably available in the anonymized dataset; as these are recognized prognostic factors, their absence is a further potential source of residual confounding. This selection bias is consistent with findings in other PDE5I studies, in which users generally exhibited different health-seeking behaviors than non-users. Additionally, reverse causation cannot be fully excluded, as healthier patients may be more likely to receive PDE5Is, while patients with advanced disease or comorbidities may be less likely to be prescribed these medications. Longitudinal randomized controlled trials are necessary to definitively test the causal impact of PDE5Is on rectal cancer survival and to differentiate drug effects from confounding behavioral factors. Furthermore, the data relies on medication purchase history, which does not confirm actual usage or adherence to PDE5I therapy. The heterogeneity of the patient population, particularly regarding ethnicity and comorbidities, may also affect generalizability. Since cause of death is not recoverable from the CHS database, we used overall survival as the primary endpoint and did not apply formal competing risk models to distinguish cancer-related from non-cancer deaths. Given the older age and comorbidity burden of this cohort, differences in non-cancer mortality between PDE5I users and non-users could have influenced the observed survival patterns. Future studies with detailed cause-of-death information and appropriate competing risk or cause-specific hazard modeling will be important to clarify the extent to which the association we observed reflects cancer-related rather than competing non-cancer mortality. Lastly, anonymization necessitated the loss of important clinical data including tumor stage, type of surgery and specifics of chemotherapy/radiotherapy, which limited our ability to comprehensively adjust for all relevant prognostic factors. The absence of key oncologic variables such as tumor stage at diagnosis, histologic grade, lymphovascular invasion, and details of primary treatment (type of surgery, neoadjuvant/adjuvant chemotherapy or radiotherapy regimens) represents an important limitation of our study. Without these data, we cannot exclude residual confounding by disease severity or treatment intensity—PDE5I users may have had, on average, earlier-stage disease, more favorable tumor biology, or received more complete curative therapy, any of which could contribute to their observed survival advantage. This unmeasured confounding likely limits the strength of causal inference and underscores the need for future studies with comprehensive clinical staging and treatment data.

## 5 Conclusion

In the current study, post-surgical PDE5I use was associated with improved overall survival among men treated for rectal cancer. This association persisted after adjustment for measured confounders and propensity-score matching. However, residual confounding, exposure misclassification, and healthy-user bias preclude causal interpretation. These hypothesis-generating findings, together with emerging mechanistic evidence, support further prospective and translational investigation of PDE5 inhibition in colorectal cancer.

## Data Availability

The datasets presented in this article are not readily available because Restrictions apply to the availability of these data. Data were obtained from CHS’s Research Room platform and cannot be provided by the authors due to ethical restrictions. Requests to access the datasets should be directed to Prof. Gil Bar-Sela, gil_ba@clalit.org.il.
