# Supplementary material for: Assessing the impact of phosphodiesterase 5 inhibitors on survival in men after treatment for rectal cancer
Source: Front Oncol. 2026 Sep 22;16:1940968. doi: 10.3389/fonc.2026.1940968 (PMC13638660; doi:10.3389/fonc.2026.1940968)
Supplement: Supplementary file 1 [file DataSheet1.docx]

Supplementary Materials: The Use of Phosphodiesterase 5 Inhibitors Improves Survival in Men after Treatment for Rectal Cancer

Myroslav Lutsyk, Jonathan Green, Igor Albitskiy and Gil Bar-Sela

**Figure S1:** Age distribution at diagnosis of PDE5I users and non-users. Age distribution at diagnosis of PDE5I users (displayed in green on the right) and non-users (displayed in blue on the left). The graphical representations were created using histograms and Kernel Density Estimation. Mean ages at diagnosis are represented by the dashed lines (non-users clearly have higher mean diagnosis age). As displayed in the graphs (and verified in the Shapiro-Wilks tests), while PDE5I users’ distribution seems to be approximately normal, non-users’ distribution seems to be more skewed.

**Table S1.** Top 6 Variables/coefficients for Standardized Cox PH Model and Random Survival Forest.

| **Standardized Cox PH** | | **Random Survival Forest** | |
| --- | --- | --- | --- |
| **Variable: Level** | **Relative Importance** | **Variable** | **Importance** |
| Ethnicity: Other | 0.261 | Recurrence | 0.0564 |
| Solid Tumor: Metastatic | 0.090 | Age at diagnosis | 0.0318 |
| Recurrence | 0.089 | CCI score | 0.030 |
| Liver disease: Moderate-Severe | 0.065 | Ethnicity | 0.025 |
| Age at diagnosis | 0.056 | Drug use | 0.016 |
| Comorbidity count | 0.045 | Comorbidity count | 0.007 |
